# Highly Charged Cellulose Nanocrystals via Electrochemical Oxidation

**DOI:** 10.1021/acs.nanolett.4c02918

**Published:** 2024-11-06

**Authors:** Neptun Yousefi, Jenna Hannonen, Lukas Fliri, Pekka Peljo, Eero Kontturi

**Affiliations:** † Department of Bioproducts and Biosystems, Aalto University, P.O. Box 16300, 00076 Aalto, Finland; ‡ Battery Materials and Technologies, Department of Mechanical and Materials Engineering, University of Turku, FI-20014 Turun yliopisto, Finland

**Keywords:** cellulose nanocrystals, electrochemical oxidation, gaseous acid, hydrolysis

## Abstract

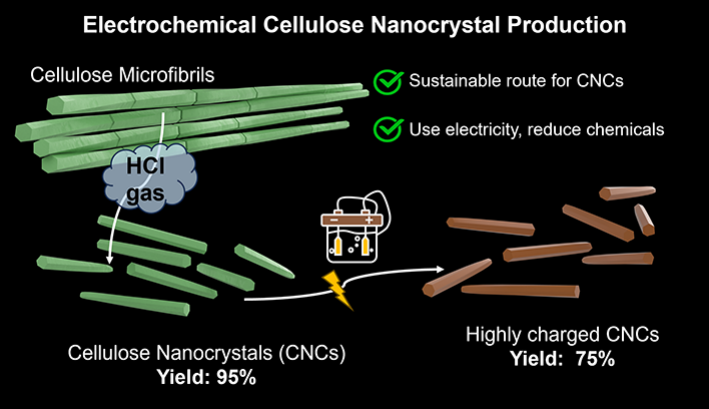

Due to their exceptional properties, cellulose nanocrystals (CNCs) have been proposed for various applications in sustainable materials science. However, state-of-the-art production methods suffer from low yields and rely on hazardous and environmentally harmful chemicals, representing a bottleneck for more widespread utilization of CNCs. In this study, we present a novel two-step approach that combines previously established HCl gas hydrolysis with electrochemical TEMPO oxidation. This unique method allows the collection of easily dispersible CNCs with high carboxylate contents in excellent overall yields of 71%. The electromediated oxidation was conducted in aqueous conditions without the usually required cocatalysts, simplifying the purification of the materials. Moreover, the proposed process is designed for facile recycling of the used reagents in both steps. To evaluate the sustainability and scalability, the environmental impact factor was calculated, and a cost analysis was conducted.

Cellulose nanocrystals (CNCs) are, in essence, short rigid rods of crystalline cellulose produced from plant-based or microbially generated filaments. CNCs are valued for their high strength and stiffness and have received increasing research interest with ambitions to exploit the peculiar properties of nanoscale materials using renewable and sustainable feedstocks. Consequently, a wide variety of potential applications have been proposed for CNCs, ranging from composites^1^ and biomedical templates^2−4^ to chiral catalyst carriers.^5,6^ Although obtained from a biobased source, the isolation of CNCs following established protocols cannot be considered as environmentally benign. In the state-of-the-art preparation method, concentrated sulfuric acid is used to hydrolyze the disordered regions of the cellulosic starting material and introduce charged sulfate half-ester moieties on the crystallite surface.^7^ The harsh conditions result in the formation of soluble (oligo)saccharidic fractions as a side product. Thus, only low yields in the order of 20–50% can be obtained.^8^ Moreover, significant amounts of contaminated sulfuric acid are generated. Alternative isolation routes have been proposed to address the associated environmental concerns, *e.g.*, involving mechanochemistry,^9,10^ electron beam irradiation,^11^ or enzymatic pathways.^12^ However, they have remained odd accounts in the literature and have not been developed further into viable green technology solutions. A genuinely sustainable pathway to CNCs is still sought and would contribute substantially to the utilization of biobased materials.

A CNC isolation method that has received attention recently is the treatment of cellulose using anhydrous HCl gas in a gas/solid system.^13−17^ As opposed to the aforementioned liquid/solid system, no solubilization can occur, resulting in almost quantitative yields. Moreover, the gaseous acid is far easier to recycle, and the purification of the product is relatively effortless. The bottleneck in the process is the lack of surface charge after the reaction. Consequently, a second modification step is required to allow for dispersion of the CNCs for further application. This has been tackled by, *e.g.*, phosphorylation,^17^ 2,2,6,6-tetramethylpiperidine 1-oxyl (TEMPO)-oxidation,^16^ and using soluble polysaccharides^18^ as dispersing agents. Several other derivatization protocols are summarized and discussed in the Supporting Information (SI) (Table S1).

TEMPO-mediated oxidation^19^ – resulting in selective carboxylation of the primary alcohol groups on the surface of CNCs–has achieved high charge and mass yields.^16^ However, the use of hypochlorite and sodium bromide as coreactants is considered expensive and problematic from an environmental perspective.^20^

Electrochemical setups were proposed for the TEMPO oxidation of other cellulose substrates, like cellulose nanofibrils^21^ or CNCs isolated from *Cladophora*.^22^ However, in the reported protocols, long reaction times were required to achieve high surface charges,^21^ or sufficient carboxylate contents could not be achieved.^22^

Here, we adapted and optimized the electrochemical TEMPO-oxidation for the preparation of CNCs after prehydrolysis with HCl gas (Figure 1). The reaction can be conducted under aqueous conditions using only the TEMPO catalyst, the recycling of which has been demonstrated on several occasions.^23,24^ Our study advances the state-of-the-art by combining HCl-gas hydrolysis with electrochemical oxidation, providing a simple approach for the preparation of highly charged CNCs. Specifically, HCl (g) hydrolyzes the noncrystalline segments in cellulose microfibrils, which is essential for CNC preparation. TEMPO-oxidation, in turn, introduces charged carboxylates on the crystallite surface, which is essential for CNC dispersion. Carboxylated CNCs produced through our electrochemical oxidation method not only achieve high surface charge, enhancing their dispersion stability in aqueous media, but they also avoid the environmental risks associated with phosphorus-based processes, such as eutrophication. Moreover, our method is more sustainable and does not require the use of harsh chemicals or additional reagents, unlike, *e.g.*, phosphorylation methods.

**Figure 1:**
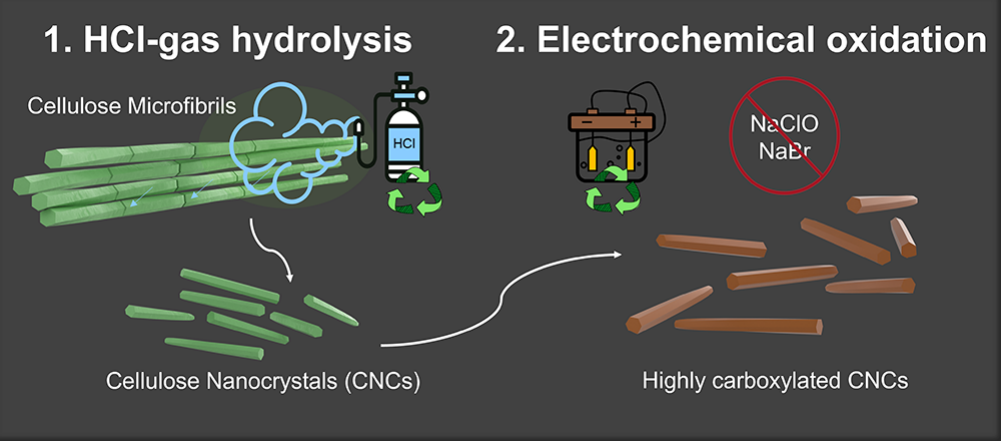
Schematic of carboxylated CNC preparation from bacterial cellulose: (1) HCl-gas hydrolysis, followed by (2) electrochemical oxidation of the hydrolyzed cellulose.

Figure 1 illustrates the route from bacterial cellulose (BC) to carboxylated CNCs. Briefly, BC was washed and dried before being weighed and placed in a custom-built reactor for hydrolysis using gaseous HCl. The hydrolysis took place overnight at 1 bar, as reported previously.^16^

After washing thoroughly, hydrolyzed BC was obtained. Gel permeation chromatography showed that the molecular weight of the hydrolyzed BC is lower than that of the untreated BC (Figure 2). The mass average molecular weight (*M*_w_) decreased from 326600 to 43400 Da for BC and hydrolyzed BC, respectively. The degree of polymerization (DP) decreased from roughly 2000 to 270 for BC and hydrolyzed BC, respectively, indicating successful hydrolysis.

**Figure 2:**
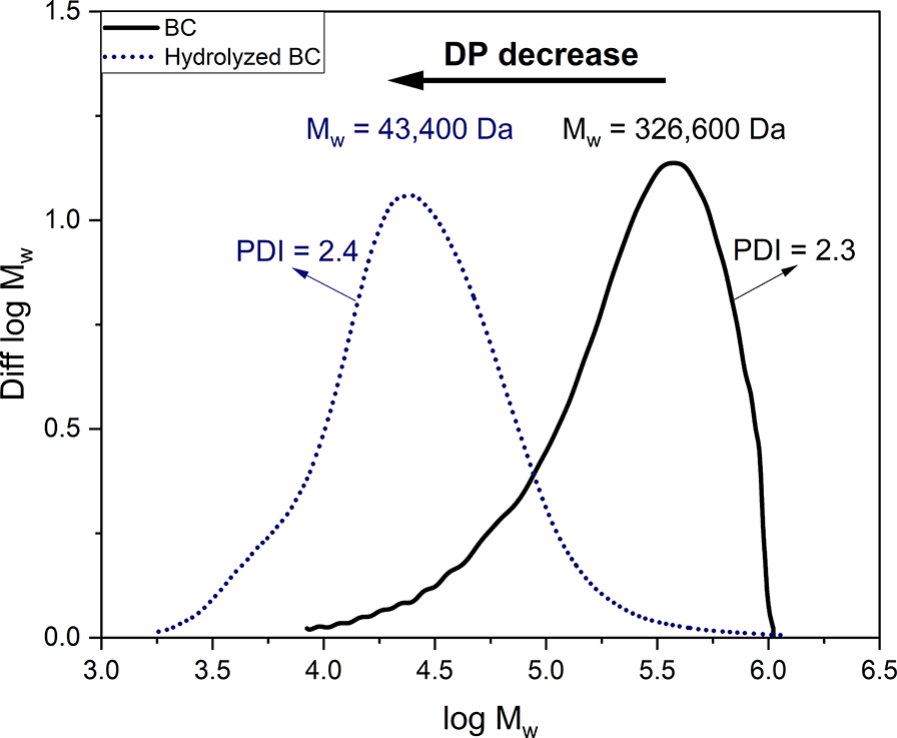
Gel permeation chromatogram of untreated bacterial cellulose and hydrolyzed bacterial cellulose.

The subsequent electrochemical oxidation was performed on a laboratory scale using 0.5 g of hydrolyzed bacterial cellulose in a 100 mL carbonate buffer solution with 2 mmol of TEMPO. The reaction occurred in a three-electrode setup consisting of a carbon foam working electrode, a titanium mesh counter electrode, and an Hg|Hg_2_SO_4_|K_2_SO_4_ reference electrode. The process was optimized for high efficiency, achieving a current efficiency of 98% with a stable potential of 0.5 V applied over 24 h. Hydrolyzed BC was electrochemically oxidized using this setup for up to 24 h at a constant potential of 0.5 V. Figure 3 shows a cyclic voltammogram (CV) obtained using our electrochemical setup containing a TEMPO catalyst in a pH 10 carbonated buffer measured against a reference electrode. The anodic peak in the CV shows the oxidation of the TEMPO radical to the *N*-oxoammonium ion, while the cathodic peak shows the reduction to the *N*-hydroxy form. Under these conditions, the reaction of the TEMPO catalyst is reversible.

**Figure 3:**
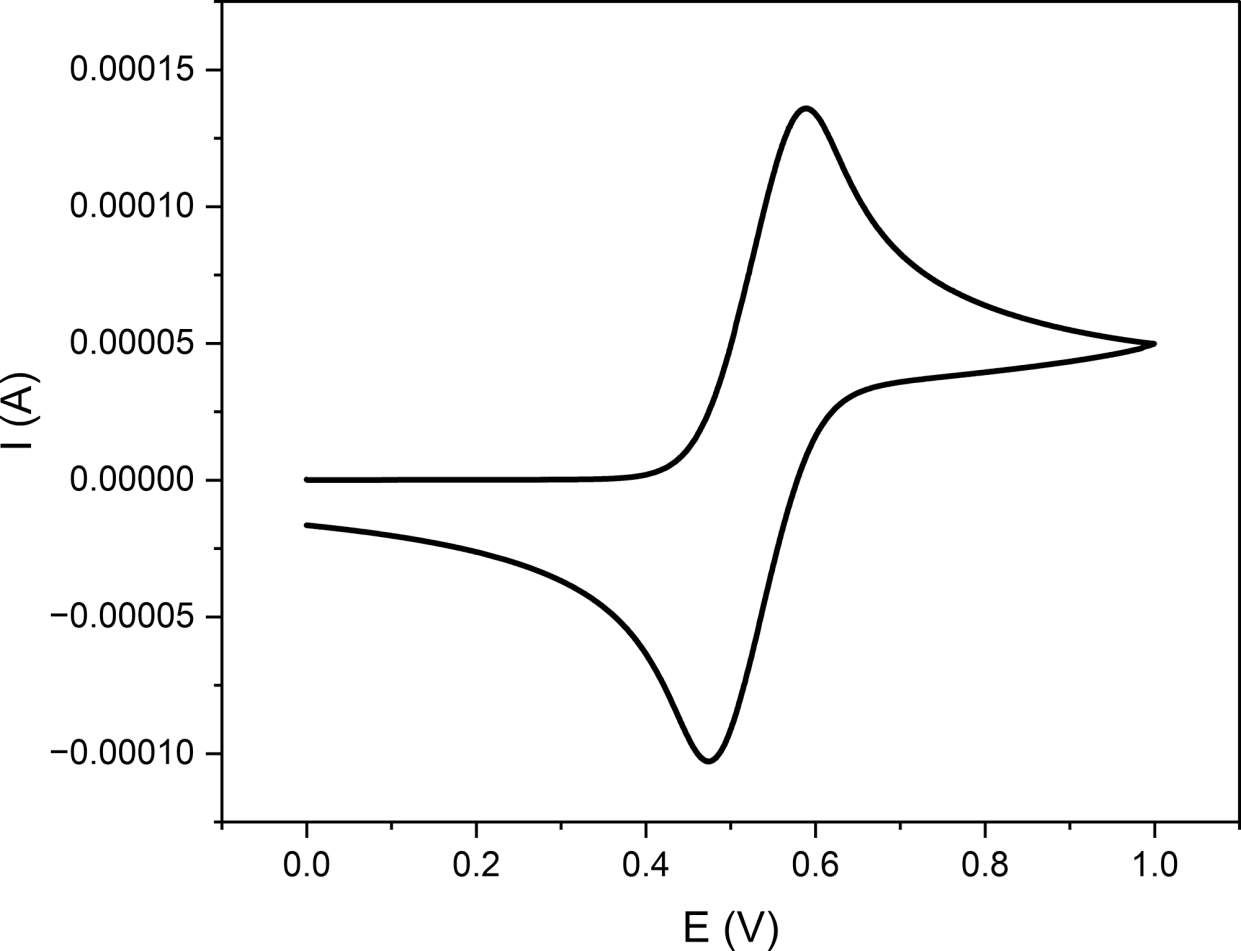
Cyclic voltammogram of TEMPO in carbonated buffer at pH 10 at a scan rate of 50 mV/s. The applied potential (*E*) was measured against an Ag|AgCl reference electrode.

We demonstrated the pH dependence of TEMPO during the electrochemical reaction (Figure S1) and its reversibility using different buffer systems (Figure S2) to identify the most efficient buffer system for our electrochemical oxidation. The use of high-surface-area electrodes reduced oxidation times from the previously reported 45 h^21^ to 12 h. Our electrochemical oxidation process is less labor-intensive than traditional TEMPO oxidation methods and enables precise and controlled oxidation. Our buffer system could also be reused twice after the initial electrochemical reaction upon removal of the newly carboxylated CNCs and the introduction of fresh substrate, which improves scalability and efficiency. Currently, we are optimizing the process to reduce the reaction time by adjusting electrode material and current settings, making it sustainable and suitable for large-scale production. More information on the system’s electrochemistry is provided in the Supporting Information (SI).

The surface charge, and hence the carboxylate content of CNCs, was determined by conductometric titration. With increasing electrochemical oxidation time, a higher carboxylate content was obtained, reaching a maximum of 1.24 ± 0.15 mmol/g after 24 h for the hydrolyzed BC (Figure 4). We also attempted the electrochemical oxidation of bacterial cellulose without prior hydrolysis, which resulted in a carboxylic acid content of 0.7 ± 0.2 mmol/g after 24 h. Therefore, hydrolysis appears to increase the accessibility of BC in CNC preparation.

**Figure 4:**
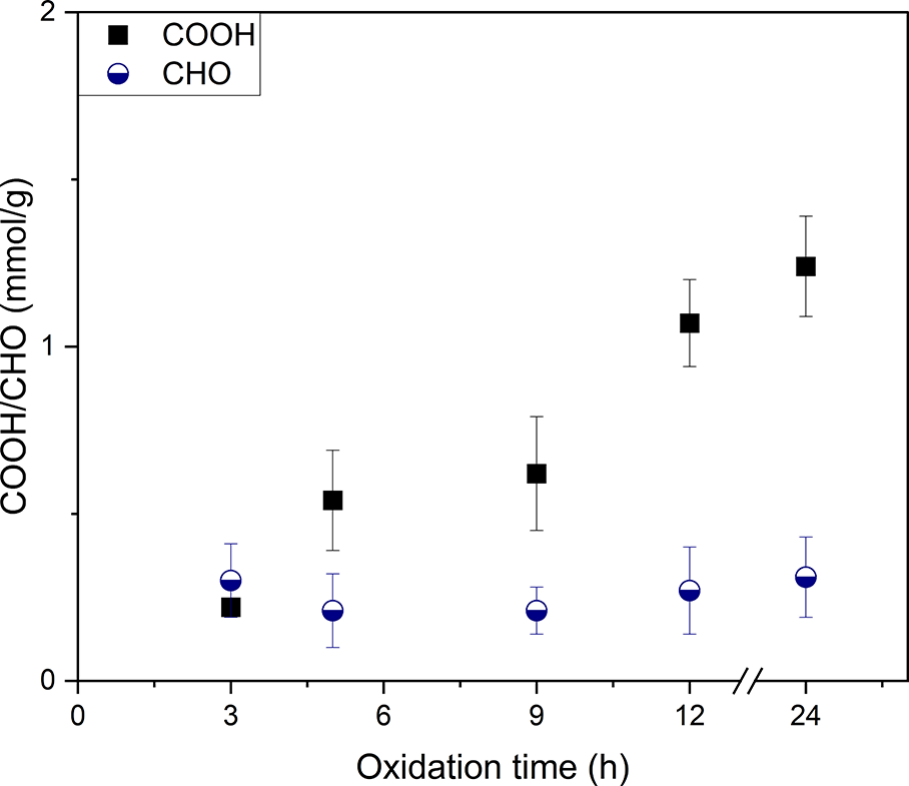
Carboxylate and aldehyde content of hydrolyzed BC after electrochemical oxidation.

As the formation of carboxylate groups proceeds via aldehyde intermediates, they were monitored by conversion to their oxime derivatives for elemental analysis, post electrochemical oxidation. This analytical protocol was adapted from procedures predominantly used in dialdehyde cellulose research.^25^ Conventional aldehyde-specific titration techniques for TEMPO-oxidized cellulose could not be performed on the small sample amounts available from the used electrochemical setup. After 3 h of electrochemical oxidation, the aldehyde content was higher than the carboxylate content, but with increasing time, the aldehyde content remained roughly the same while the carboxylate content increased (Figure 4). Both aldehyde and carboxylate content were determined at least in triplicate for each oxidation time. We demonstrated that our method is effective for other cellulose sources, as evidenced by the successful oxidation of a cotton linter (1.35 ± 0.21 mmol/g) under the same conditions.

Traditional TEMPO oxidation (TEMPO/NaBr/NaClO system) for BC provided an aldehyde and carboxylate content of 0.1 and 1.05 mmol/g, respectively.^26^ In our electrochemical oxidation, we achieved a carboxylate content of 1.07 ± 0.13 mmol/g, which is consistent with values reported in the literature for BC after 12 h. Our findings correlate somewhat with previously reported carboxylate contents of *Cladophora* also produced using electromediated TEMPO-oxidations, which were 0.595 and 0.599 mmol/g after 1 or 3 days, respectively.^22^ The smaller surface charge in *Cladophora* is justified by the larger crystal size present in *Cladophora* CNCs.

The CNC yield after electrochemical oxidation was 75% across all oxidation times, indicating that no considerable solubilization of highly charged chains occurred. The remaining yield loss is predominantly assignable to handling losses during workup. Pääkkönen *et al*. reached a similar CNC yield of 80% using HCl gas hydrolysis and conventional TEMPO oxidation.^16^ Other processes using different cellulose sources typically reach CNC yields of around 15–50%, including hydrolysis using sulfuric acid (yields 15–50%),^7^ and esterification (yield 25%).^27^ Details on our CNC yield calculations can be found in the SI.

We evaluated whether the surface modification of our CNCs can be considered quantitative. Lacking a model crystallite, we used cotton linters as a reference, as they share a similar crystallite dimension to our CNCs.^28^ Using this model, we estimated a complete conversion of our CNCs, signifying that all surface-accessible sites were oxidized to carboxylic acid (detailed calculations are available in the SI).^29,30^ The full conversion should be interpreted cautiously, as it may be influenced by the use of an inadequate crystallite model and the potential for overoxidation on crystallite end sites.^31^

The length and width of the electrochemically oxidized CNCs were investigated using atomic force microscopy (AFM, Figure 5a and Figure S5) and transmission electron microscopy (TEM, Figure S3), respectively.

**Figure 5:**
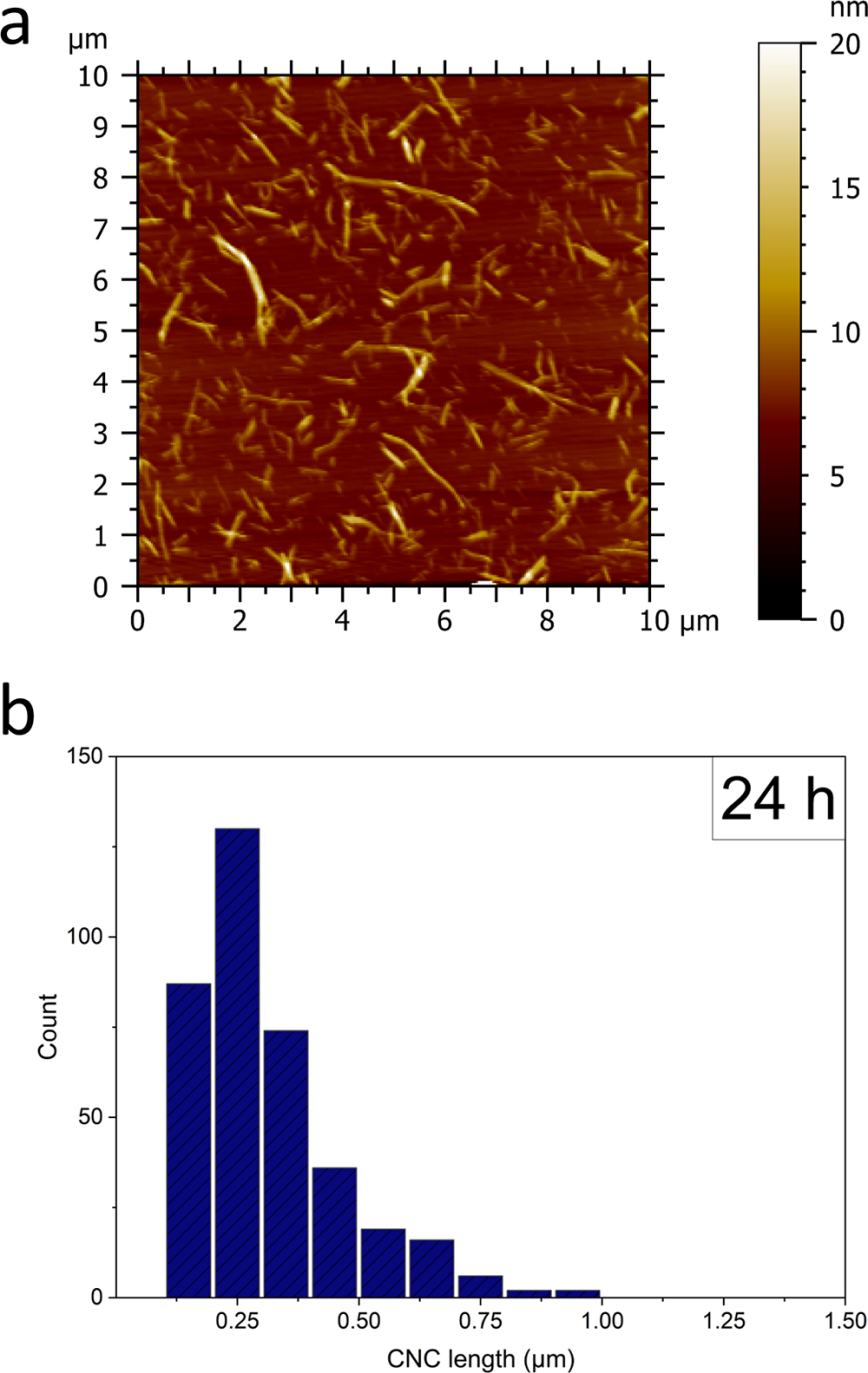
(a) Selected AFM images after 24 h of electrochemical oxidation. (b) Length distribution of hydrolyzed BC after 24 h of electrochemical oxidation.

After 9 h of electrochemical oxidation, the average CNC length was 353 ± 277 nm (Figure S5). After 12 h, the average CNC length remained similar (349 ± 205 nm; Figure S5), but after 24 h had decreased slightly to 313 ± 155 nm (Figure 5b). We determined the width of CNCs to be 13 ± 6 nm after 24 h of electrochemical oxidation (Figure S4). Vasconcelos *et al*. produced CNCs from BC under different hydrolysis conditions, showing lengths from 200 to 1000 nm (average 622 ± 100 nm) and widths ranging from 16 to 50 nm (average 34 ± 14 nm),^32^ in good agreement with our findings. Another report on CNCs from BC showed average lengths and widths of 855 and 17 nm, respectively.^28^ However, no standard deviation was provided, probably due to a small available sample pool. CNCs produced from BC using conventional TEMPO oxidation were reported to have lengths of 100 to 300 nm (average 170 nm).^16^ It should be noted, however, that only a small number of CNCs were measured in that study. Full length and width distributions and AFM and TEM images of all samples can be found in the SI (Figure S3–5). Essentially, the lower the charge of the CNCs, the more difficult it is to disperse them. The dispersion ability is seen in Figure S5a, where after 9 h of electrochemical oxidation, the samples had to be diluted by a factor of 10 to avoid agglomeration. Such dilution was not required after 12 or 24 h of electrochemical oxidation.

The entire process was examined by calculating its environmental impact factor (*E* factor). A higher *E* factor means more waste and, consequently, more significant adverse environmental impact. The ideal *E* factor is zero. More recently, the inventor of the *E* factor concept suggested using simple *E* factors (sEF) and complete E factors (cEF), depending on the process’s development stage.^33^ The sEF does not consider solvents and water and hence assumes recycling of solvents. In contrast, the cEF accounts for all process materials, including solvents and water, assuming no recycling and is more appropriate for total waste stream analysis. We have calculated both sEF and cEF of our process. Both the sEF and cEF for our gaseous hydrolysis are 2, as no solvents or water was used during the hydrolysis. The sEF of the electrochemical oxidation is 2, whereas the cEF is 134. As the TEMPO catalyst and the buffer solution can be reused,^23,24^ our entire process has a simple *E* factor of 2 for both HCl gas hydrolysis and electrochemical oxidation. Equations and calculations of sEF and cEF can be found in the SI.

A cost analysis of our CNC production process was conducted. All details on the calculations, including energy consumption, can be found in the SI (Tables S2–S5). We want to emphasize that the absolute cost of these materials is exclusively for comparative purposes and only considers lab-scale production.

Our method distinguishes itself with a notably high yield, demonstrating its potential for efficient scalability in cellulose nanoparticle production compared to existing literature. We emphasize responsible practices by implementing stringent measures to minimize the use of harmful substances. Our approach contributes to the production of oxidized cellulose by reducing chemicals such as NaClO, NaBr, and NaOH, underscoring our commitment to responsible and conscientious nanocellulose production.

We developed an effective and simple route to CNCs in high overall yields (71%) using electrochemical oxidation with a TEMPO catalyst. Our route produced highly charged CNCs containing 1.2 mmol/g of carboxylate groups. The process was conducted in an aqueous buffer and did not require the use of hypochlorite. The resulting CNCs had a length and width of roughly 313 ± 155 and 13 ± 6 nm, respectively. Overall, our process provides a new electrochemically driven route to CNCs without excessive chemical waste generation compared to conventional alternatives.

## Supporting Information Available

Experimental details, materials, methods, and results, including CVs, AFM and TEM images, cost, and *E* factor calculations (PDF)

## Abbreviations

CNC: cellulose nanocrystal
TEMPO: 2,2,6,6-tetramethylpiperidine 1-oxyl
BC: bacterial cellulose
*M*_w_: mass average molecular weight
DP: degree of polymerization
PDI: polydispersity index
CV: cyclic voltammogram
*E* factor: environmental impact factor
sEF: simple *E* factor
cEF: complete *E* factor
