# Supplementary material for: Highly Charged Cellulose Nanocrystals via Electrochemical Oxidation
Source: Nano Lett. 2024 Nov 6;24(46):14610–4. doi: 10.1021/acs.nanolett.4c02918 (PMC11583315; doi:10.1021/acs.nanolett.4c02918)
Supplement: Supplementary file 1 — nl4c02918_si_001.pdf [file nl4c02918_si_001.pdf]

Supporting information:

Highly Charged Cellulose Nanocrystals via Electrochemical Oxidation

Neptun Yousefi,[*a] Jenna Hannonen,[b] Lukas Fliri,[a] Pekka Peljo,[b] and Eero Kontturi,*[*a]*

[a] Department of Bioproducts and Biosystems, Aalto University, P.O. Box 16300, 00076 Aalto, Finland. E-mail: neptun.yousefi@aalto.fi, eero.kontturi@aalto.fi

[b] Battery Materials and Technologies, Department of Mechanical and Materials Engineering, University of Turku, FI-20014 Turun yliopisto, Finland.

1 Experiment details

1.1 Materials

Bacterial cellulose (BC) was obtained from commercial sources (Chaokoh Nata De Coco, Theppadungporn Coconut Co., Ltd, Thailand). The purification protocol involved a meticulous process, commencing with thoroughly washing BC cubes stored in coconut gel. Subsequently, alkali extraction was performed using 0.1 M NaOH for 3 h at 85 °C. Following the extraction, the BC cubes underwent washing with pure water. The final extracted product was freeze-dried using an Edwards Micro Modulyo Freeze Dryer (Crawley, England). These purification steps ensured a high-quality cellulose source with a dry matter content exceeding 90%. The bacterial cellulose in our study was subjected to freeze-drying without pre-freezing, a method explored previously for its impact on BC's surface characteristics. This approach influences surface area, pore volume, and pore size, as highlighted in our earlier investigation.[1] 2,2,6,6-tetramethylpiperidinyloxyl (TEMPO), NaOH, HCl, NH_2OH HCl, NaOAc and HOAc were purchased from Sigma Aldrich. Na_2CO_3, $NaHCO_3$ and NaCl were acquired from Fluka. All chemicals were used as received. Throughout the study, ultrapure water with a resistivity of

18.2 MΩ·cm from a Millipore®Synergy® purification unit was used. Carbon foam (Carbon Vitreous - 3000C), a working electrode, was purchased from Goodfellow Cambridge.

1.2 Amperometric system/ Electromediated oxidation

A pH 10 Na_2CO_3/ $NaHCO_3$ buffer with a concentration of 0.1 M was prepared. TEMPO (2 mmol) was added to 100 mL of buffer and stirred using a magnetic stirrer for approximately 10 min before adding the cellulose source (0.5 g). We used hydrolyzed bacterial cellulose. We conducted cyclic voltammetry using Gamry Reference 600+ potentiostat and a three-electrode setup, including a glassy carbon working electrode (WE), a Pt counter electrode (CE) and an Ag|AgCl reference electrode (RE). The electrochemical oxidation was performed using a potentiostat (Ivium technologies), equipped with an electrochemical cell, which featured three electrodes: A Hg|Hg_2SO_4|K_2SO_4 RE, a titanium mesh CE (3x5 cm, GuangYi Store, China) and a reticulated vitreous carbon foam WE (2x3 cm, 24 pores per cm, Sigma-Aldrich). A static potential of 0.5 V was applied. The oxidation times were 3, 5, 9, 12 and 24 h. Our TEMPO oxidation process attains a commendable current efficiency of 98%, consistent with established values in the literature,[2] highlighting the effectiveness of our method in achieving high oxidation efficiency.

In our study, we have experimentally investigated the influence of pH on TEMPO electrochemical behavior. As shown in **Figure S1** below, we measured TEMPO at different pH values, clearly illustrating a significant reduction in current at pH 7. Furthermore, we conducted measurements of a TEMPO derivative (4-OH-TEMPO) under various pH conditions, revealing instances where the reaction is not fully reversible depending on the specific conditions (**Figure**

S2). These findings provide particular insights into the pH sensitivity of the electrochemical system, contributing to a nuanced understanding of the underlying chemical and electrochemical processes involving TEMPO.

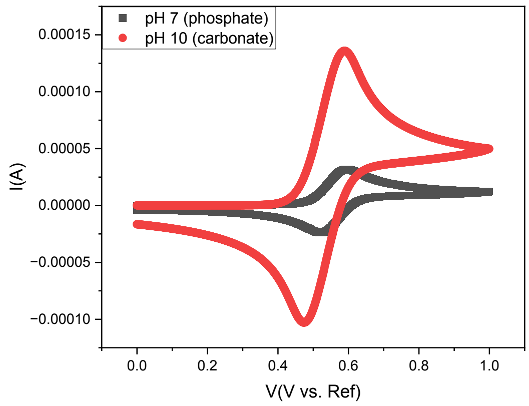

Figure S1. Cyclic voltammogram (CV) of TEMPO (2 mmol) at different pH using phosphate or carbonate buffer, both at a concentration of 0.1 M.

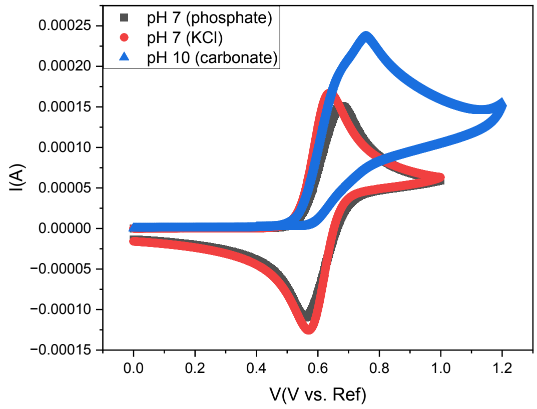

Figure S2. CVs of 4-OH-TEMPO (2 mmol) using different buffers. The concentration of KCl was 1 M, while phosphate and carbonate buffers had a concentration of 0.1 M.

1.3 Gel permeation chromatography of BC and hydrolyzed BC

The apparatus comprises a Dionex Ultimate 3000 HPLC module, a Shodex DRI (RI-101) detector, and a Viscotek/Malvern SEC/MALS 20 multi-angle light scattering (MALS) detector. We utilized the following columns Agilent PLgel MIXED-A (x 4) and a flow rate of 0.75 mL/min. We used an injection volume of 100 μL. Detector constants (MALS and DRI) were determined using a narrow polystyrene sample (Mw = 96 000 g/mol, Đ = 1.04) dissolved in 0.9% LiCl in *N*-*N*-dimethylacetamide (DMAc). A broad polystyrene sample (Mw = 248 000 g/mol, Đ = 1.73) was used to test the detector calibration. The ∂n/∂c value of 0.136 mL/g was used for celluloses in 0.9% LiCl in DMAc.[3] Bacterial cellulose and hydrolyzed bacterial cellulose (50±5 mg) were soaked in MiliQ water (5 mL) overnight. The next day, the water was removed, and the cellulose was washed with 2 mL acetone. Then, the cellulose was soaked in 4 mL acetone for 5 h. The final activation step was adding 4 mL of pure DMAc to the cellulose samples left for two days. After removing DMAc, 5 mL of LiCL/DMAc (10 g/L) was added, stirring the mixture overnight. The cellulose was dissolved entirely at this point. 0.50 mL of the sample was diluted with 4.5 mL of pure DMAc and stirred well (resulting in a concentration of 1.0 mg/mL) before the dissolved and diluted samples were filtered into vials using 0.2 μm syringe filters.

1.4 Determination of carboxylic groups

After the electrochemical oxidation, the cellulose suspension was stirred for 1 h to ensure that all $TEMPO^+$ had reacted before removing the TEMPO-containing phase by centrifugation and washing with water. The cellulose was protonated using 0.1 M HCl overnight (pH~2). The next day, the cellulose was washed with pure water to reach a neutral pH. After determining the dry matter content, a minimum of 300 mg of cellulose was used for the conductometric

measurement. The wet, washed cellulose sample (a minimum of 300 mg of cellulose when dried) was combined with 500 mL of degassed Milli-Q water and 0.5 mL of a 0.5 M NaCl solution. The mixture underwent acidification with 1 mL of a 0.1 M HCl solution and subsequent titration using a 0.1 M NaOH solution at a 0.1 mL/min rate. The value given for COOH content is an average of at least three measurements. The carboxylate content was determined by conductometric titration following SCAN-CM 65:02 using an automatic titrator (Metrohm 751 GPD Titrino) and software (Tiamo 1.21.).

1.5 Characterization of aldehyde groups

For the transformation of the aldehyde moieties, at least 20 mg of Tempo-oxidized cellulose gel (dry matter content) was added to 10 mL of a stock solution of hydroxylamine hydrochloride (0.1 M) in a pH 4 acetate buffer. The suspension was stirred in a sealed reaction vessel overnight (20-24 h) before being separated, purified by a centrifugation/washing (H_2O) protocol, and thoroughly freeze-dried. The aldehyde content was calculated from the nitrogen values obtained in elemental analysis using the simplified Equation 1:

$$CHO\left[\frac{\text{mmol}}{\text{g}}\right] = N\left[\frac{\text{mmol}}{\text{g}}\right] = \frac{N\,[wt\%]}{100} * \frac{1}{M_N\left[\frac{g}{mol}\right]} * 1000 \quad (1)$$

The adaptation of a more elaborate equation was tested considering the changes in the chemical composition introduced to the polymer by the TEMPO oxidation (COOH and COONa groups) and subsequent oximation. However, the expected error was relatively insignificant given the similar molecular weights of the modified units and the anhydroglucose units (AGUs).

Elemental Analysis (EA) was performed by combustion EA on a Thermo Flash Smart CHNSO Elemental Analyzer. For sample preparation, 1-3 mg of material was accurately weighed into

tin foil cups. Cellulosic samples were thoroughly freeze-dried before measurement. The device was calibrated by a linear calibration using 2,5-Bis(5-tert-butyl-2-benzo-oxazol-2-yl) thiophene (BBOT) as the standard. All measurements were done at least in duplicate and averaged. *C*, *H*, *N* and *S* were directly analyzed, and calculations for the aldehyde content were based on the nitrogen values.

1.6 Morphology of CNCs determined by atomic force microscopy

A CNC suspension of 0.1 g/L was prepared from each reaction time. Samples with shorter electrochemical treatment were diluted 10 times to prevent agglomerations. The silicon wafers were rinsed with acetone and deionized water before being submerged in a polyethyleneimine (PEI) solution (3.5%, Mw=2000-4000 g/mol) for 15 min. The PEI-coated wafer was cleansed gently with deionized water and dried. A spin coater (Laurell Technologies WS-650SX-6NPP/LITE) operating at 4000 rpm covered the wafer using 30 µL CNC dispersion. In tapping mode, the silicon wafers were imaged using an atomic force microscope (AFM, Bruker Multimode 8). Cantilevers (MirkoMarsch HQ:NSC15/AI BS) had a force constant and resonance frequency of 40 N/m and 325 kHz, respectively. The length of the individual particles was analyzed using the Mountains 9 software. Overlapping particles were not considered in the length analyses. 6 AFM images per oxidation time were used to determine the average CNC length distribution.

1.7 CNC width characterization using transmission electron microscopy

The width of the cellulose nanocrystals was observed with a transmission electron microscope (TEM, JEOL JEM-3200FSC) at 300 kV FEG with an in-column energy filter (omega). The

carbon grid was plasma oxidized, and the sample (3 μL) was sonicated for 5 min directly before dip-coating. The suspension used had a concentration of 0.5 g/L.

1.8 HCl-hydrolysis and yields

The cellulose hydrolysis using gaseous HCl was conducted in a purpose-built reactor designed for safety and precision. The reactor, featuring a Duran pressure plus bottle with a pressure range of −1 to 1.5 bar, ensured controlled HCl gas additions. The default HCl gas pressure was set through a degassing process, prioritizing safety measures. Coupling valves enabled swift detachment and attachment, facilitating efficient gas handling while maintaining system integrity.

After adding HCl gas, the sample bottle was safely detached for further processing. Gas lines made of PTFE were systematically flushed with compressed air and nitrogen to ensure the removal of any residual HCl. Control over gas flows was achieved using dry solenoid valves, emphasizing precision in the hydrolysis process. The 24 h hydrolysis reaction is conducted at a stable pressure of 1 bar.

Our processing design comprises two key steps: HCl-gas hydrolysis and electromediated TEMPO oxidation. The hydrolysis step after washing had an impressive yield of 95%. In the second step, electromediated TEMPO oxidation, we achieved a yield of 75%. This yield is attributed to the wet condition of our material, making product loss more feasible. Despite this, our overall process attained a remarkable total yield of 71%, a noteworthy achievement compared to current industry standards.

2 Results and Discussion

2.1 Cellulose nanocrystal oxidation pathways

Table S1. A short overview of cellulose nanocrystal oxidation.

References	Feedstocks	Main chemicals	Product	By-products	Waste
Our work	Bacterial cellulose	HCl-gas, TEMPO, carbonate buffer, cellulose	Carboxylated cellulose nanocrystals	Aldehydes	HCl-gas and TEMPO are recyclable
Sulfuric acid hydrolysis[4]	Cellulose	Concentrated sulfuric acid	Cellulose nanocrystals	Unwanted Functionalized cellulose, glucose,	No reuse/recycling of strong acid, high waste produced
TEMPO oxidation[5]	Linter cellulose and softwood bleached kraft pulp	TEMPO, NaBr, large quantities: NaOH & NaClO	Carboxylated cellulose	Aldehydes, unwanted decrease in DP	Primary oxidant: NaClO, detrimental to environment, no reuse of catalyst (TEMPO or NaBr)
Oxidation[6]	Softwood pulp	Sodium periodate, NaClO	Carboxylated cellulose	White fibrous precipitation fraction after oxidation & white precipitation: MCCs and NCCs	Two-step oxidation process; harsh chemicals chain opening of cellulose
Oxidation[7]	MCC	Sodium periodate, hexane, hexylamine	Cellulose nanocrystals	Aldehydes and carboxylated cellulose	Highly toxic environment; chain opening of cellulose
Oxidation[8]	hardwood bleached kraft pulp	TEMPO/NaClO /$NaClO_2$, at 60 C in H_2O	Carboxylated cellulose	Fewer side reactions compared to the usual TEMPO system	More $NaClO_2$ used to activate TEMPO
Oxidation[9]	Softwood and hardwood bleached kraft pulps	4-AcNH-TEMPO/NaClO /$NaClO_2$ at 40 C in acidic conditions pH 4.8	Carboxylated Cellulose		More $NaClO_2$ used to activate TEMPO

2.2 Cost estimation

The estimated cost per gram of oxidized cellulose via electrochemical oxidation is calculated. The estimated materials costs were calculated using the reagents required for hydrolysis and oxidation. For all calculations, the lowest unit price for solids or 1 L (liquids) found on Merck (www.sigmaaldrich.com) as of February 27th, 2024, was used, and the price was adjusted to adhere to the amount needed for oxidation. A minimum reagent purity of ≥98% was required. Work-up steps, such as washing, were excluded from the calculations.

Regarding electrochemistry, we considered current electricity costs in Finland (Table S5) and calculated them under our given conditions. We want to emphasize that the absolute cost of these materials is exclusively for comparative purposes and only considers lab-scale production.

Table S1. Chemicals used in this work for hydrolysis and electrochemical oxidation.

Sigma-Aldrich (Link: https://www.sigmaaldrich.com/AT/de)						
**Product number**	**Purity**	**CAS**	**Name**	**€**	**Amount**	**Unit**
214000	98%	2564-83-2	TEMPO	148	25	g
S6014-1KG	99.7%	144-55-8	NaHCO3	62.4	1000	g
223530-1KG	99.5%	497-19-8	Na2CO3	81.7	1000	g

Table S2. Cost estimation of HCl gas used in this work.

**All in gas (Link: https://all-in-gas.com/en)**				
**Order number**	**Name**	**€**	**Amount**	**Unit**
534002	HCl (gas)	220.15	34	L

Table S3. Cost estimation for 1 g of oxidized cellulose

**This work**	**used/g or mL**	**€/g or mL**	**tot. cost**
TEMPO	0.31	5.92	1.85
$NaHCO_3$	0.38	0.06	0.02
Na_2CO_3	0.57	0.08	0.05
HCl	2.00	0.01	0.02
			**1.94**

For hydrolysis, 2 mL of HCl gas was used; for electrochemical oxidation, 2 mmol of TEMPO was used in 100 mL of buffer solution. The total estimated cost of the chemicals used was 1.94 €.

Table S4. Electricity use for electrochemical oxidation of 24 h.

**Electricity for electrochemical oxidation (EO)**			
Electricity cost		Estimated EO cost	
**0.2383**	€/kWh	0.000035	kW
**Conditions of EO**		0.00084	kWh
**0.5**	V	0.0002	€
**0.07**	A		
**0.035**	W		
**24**	h	reaction time	

As our electrochemical oxidation was performed at 0.5 V and 70 mA, the power required was 0.035 W. Considering that 1 kWh costs 0.2383 € in Finland and our reaction takes 24 h, the

cost of electricity is 0.0002 € for 1 g of cellulose. Our total cost for one gram of oxidized CNCs is 1.94 €.

2.3 Physical characterization of CNCs by TEM and SEM

Figure S3 shows a representative TEM image and **Figure S4** shows the CNC width distribution of hydrolyzed BC after 24 h of electrochemical oxidation.

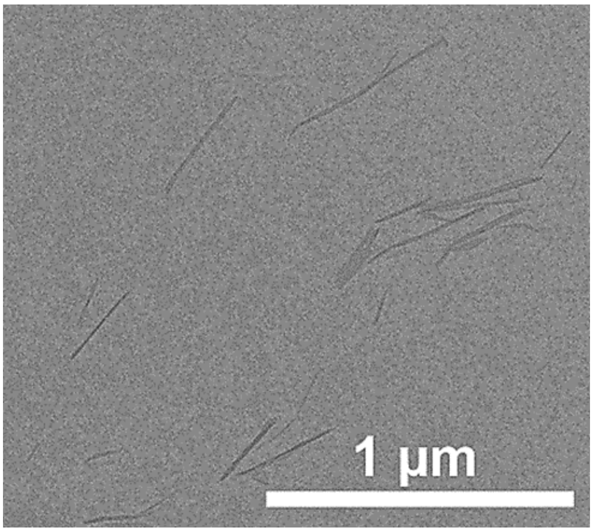

Figure S3. TEM image of CNC width after 24 h of electrochemical oxidation.

Figure S5a shows selected AFM images after 12, 9, 5 and 3 h of electrochemical oxidation. Note that the scale for the AFM images is not the same. **Figure S5b** shows the CNC length distribution after 12, 9, 5 and 3 h of electrochemical oxidation. The CNCs are slightly longer after 5 h, being 375 nm. Note that these samples had to be highly diluted as agglomeration was rather common. The sample pool was n=171, n=142, n=190, n=371 and n=371 for 3, 5, 9, 12 and 24 h, respectively.

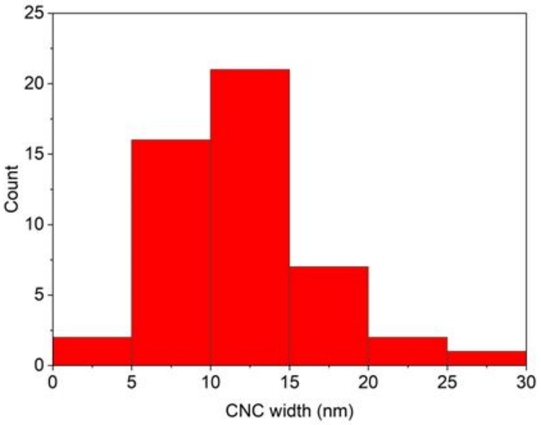

Figure S4. Width distribution of CNCs after 24 h of electrochemical oxidation.

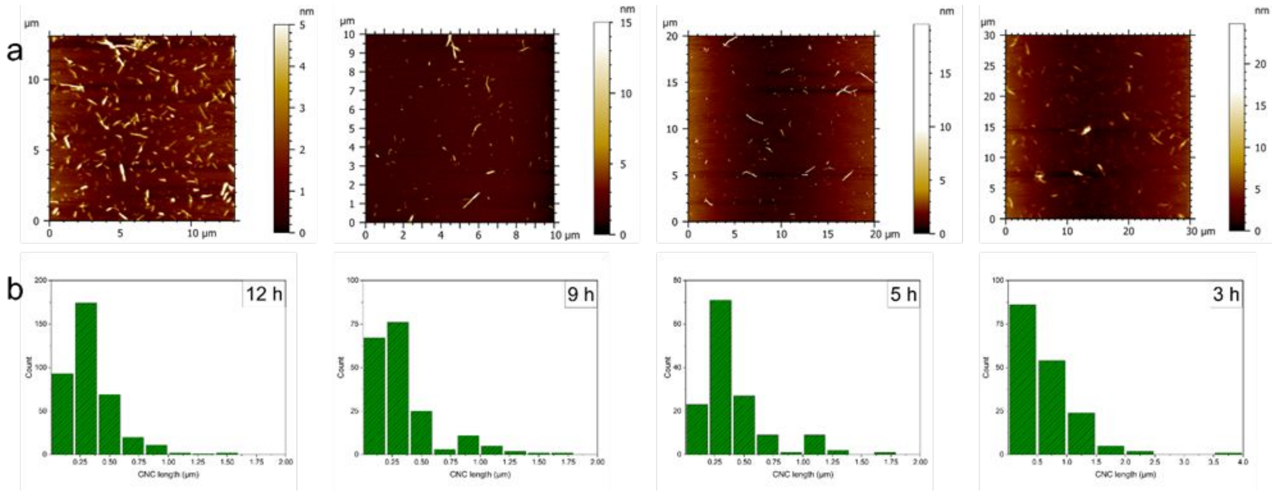

Figure S5. (a) AFM images after 12, 9, 5 and 3 h of electrochemical oxidation. (b) CNC length distributions of hydrolyzed BC after 12, 9, 5 and 3 h of electrochemical oxidation.

2.4 Carboxylate group quantification

The highest surface charge we obtained after 24 h of electrochemical oxidation was 1.24 mmol/g COOH.

Anhydroglucose has a molecular weight of 162 g/mol per unit, corresponding to 6.17 mmol for 1 g of cellulose. In one cellulose crystallite of a cotton linter fiber, the total number of chains

is 144, with 38 accessible surface chains.[10] We assumed that the C6-OH group of every second surface AGU can be converted to carboxylic acid. That means 38 AGUs out of 144 are at the surface and 50% of those (19 AGUs) can be converted to carboxylic acid (13.2%). We obtained 0.814 mmol/g (0.132*6.17 mmol/g) of surface AGU accessible to carboxylation. The ratio of our carboxylated CNCs (1.24 mmol/g after 24 h of oxidation) and the surface accessible groups is 1.52 (1.24/ 0.814). We achieve a conversion exceeding 100% due to our assumption based on accessible surface AGU. 18 or 24-chain model,[11] do not apply given the notably smaller crystalline size compared to the BC crystalline size present in this work. The difference arises from an insufficient BC crystallite model or potential overoxidation at crystallite end sites. Studies such as the one by Saito et al.[12] and subsequent works[13] have demonstrated that when oxidized or hydrolyzed (to the LODP), the ribbon-like BC microfibrils are broken to crystallites of ca. 6-7 nm in width, similar to the size of cotton. This observation suggests that the design process using bacterial cellulose can be extrapolated to other sources of cellulose with similar characteristics. Saito's research has also shown the effectiveness of TEMPO oxidation for various sources of cellulose. Furthermore, the literature indicates that bacterial cellulose, subjected to further nanocellulose fragmentation, shares a similar size with cotton. Notably, the microfibrils of bacterial cellulose form agglomerates of individual crystals, as illustrated in Fink's work.[14]

These citations from the literature support our assumption that the design process can be applied to other sources of cellulose with crystal sizes comparable to cotton. Providing new sets of data for different crystallite sizes would multiply this study's experimental matrix, which is, therefore, outside the scope of this communication.

2.5 E factor calculations of the entire process

To calculate the simple *E* factors[15] (sEF, Equation 2) and complete *E* factors[15] (cEF, Equation 3) of our processes, the following equations were used:

$$\boldsymbol{sEF} = \frac{\sum(\boldsymbol{raw\ materials})+\sum(\boldsymbol{reagents})-\boldsymbol{m}(\boldsymbol{product})}{\boldsymbol{m}(\boldsymbol{product})} \quad (2)$$

$$\boldsymbol{cEF} = \frac{\sum(\boldsymbol{raw\ materials})+\sum(\boldsymbol{reagents})+\sum(\boldsymbol{solvents})+\boldsymbol{m}(\boldsymbol{water})-\boldsymbol{m}(\boldsymbol{product})}{\boldsymbol{m}(\boldsymbol{product})} \quad (3)$$

For the HCl gas hydrolysis, 2 mL of gas is used and the product yield is 95%. This gives a sEF and cEF of 2. For the electrochemical oxidation, we have a 100 mL buffer (containing 0.3876 and 0.5709 g $NaHCO_3$ and Na_2CO_3, respectively), 2 mmol TEMPO and an oxidation product yield of 75%. This gives a sEF of 2 and cEF of 134.

References

(1) Pääkkönen, T.; Spiliopoulos, P.; Knuts, A.; Nieminen, K.; Johansson, L.-S.; Enqvist, E.; Kontturi, E. From vapour to gas: optimising cellulose degradation with gaseous HCl. *React.. Chem. Eng.* **2018**, *3* (3), 312-318. DOI: 10.1039/C7RE00215G.

(2) Milshtein, J. D.; Barton, J. L.; Darling, R. M.; Brushett, F. R. 4-acetamido-2,2,6,6-tetramethylpiperidine-1-oxyl as a model organic redox active compound for nonaqueous flow batteries. *J. Power Sources* **2016**, *327*, 151-159. DOI: 10.1016/j.jpowsour.2016.06.125.

(3) Potthast, A.; Radosta, S.; Saake, B.; Lebioda, S.; Heinze, T.; Henniges, U.; Isogai, A.; Koschella, A.; Kosma, P.; Rosenau, T.; et al. Comparison testing of methods for gel permeation chromatography of cellulose: coming closer to a standard protocol. *Cellulose* **2015**, *22* (3), 1591-1613. DOI: 10.1007/s10570-015-0586-2.

(4) Dong, X. M.; Revol, J.-F.; Gray, D. G. Effect of microcrystallite preparation conditions on the formation of colloid crystals of cellulose. *Cellulose* **1998**, *5* (1), 19-32. DOI: 10.1023/A:1009260511939.

(5) Isogai, A.; Kato, Y. Preparation of Polyuronic Acid from Cellulose by TEMPO-mediated Oxidation. *Cellulose* **1998**, *5* (3), 153-164. DOI: 10.1023/A:1009208603673.

(6) Yang, H.; Alam, M. N.; van de Ven, T. G. M. Highly charged nanocrystalline cellulose and dicarboxylated cellulose from periodate and chlorite oxidized cellulose fibers. *Cellulose* **2013**, *20* (4), 1865-1875. DOI: 10.1007/s10570-013-9966-7.

(7) Liu, P.; Pang, B.; Tian, L.; Schäfer, T.; Gutmann, T.; Liu, H.; Volkert, C. A.; Buntkowsky, G.; Zhang, K. Efficient, Self-Terminating Isolation of Cellulose Nanocrystals through Periodate Oxidation in Pickering Emulsions. *ChemSusChem* **2018**, *11* (20), 3581-3585. DOI: 10.1002/cssc.201801678.

(8) Saito, T.; Hirota, M.; Tamura, N.; Kimura, S.; Fukuzumi, H.; Heux, L.; Isogai, A. Individualization of Nano-Sized Plant Cellulose Fibrils by Direct Surface Carboxylation Using TEMPO Catalyst under Neutral Conditions. *Biomacromolecules* **2009**, *10* (7), 1992-1996. DOI: 10.1021/bm900414t.

(9) Tanaka, R.; Saito, T.; Isogai, A. Cellulose nanofibrils prepared from softwood cellulose by TEMPO/NaClO/NaClO2 systems in water at pH 4.8 or 6.8. *Int. J. Biol. Macromol.* **2012**, *51* (3), 228-234. DOI: 10.1016/j.ijbiomac.2012.05.016.

(10) Elazzouzi-Hafraoui, S.; Nishiyama, Y.; Putaux, J.-L.; Heux, L.; Dubreuil, F.; Rochas, C. The Shape and Size Distribution of Crystalline Nanoparticles Prepared by Acid Hydrolysis of Native Cellulose. *Biomacromolecules* **2008**, *9* (1), 57-65. DOI: 10.1021/bm700769p.

(11) Koso, T.; Beaumont, M.; Tardy, B. L.; Rico del Cerro, D.; Eyley, S.; Thielemans, W.; Rojas, O. J.; Kilpeläinen, I.; King, A. W. T. Highly regioselective surface acetylation of cellulose and shaped cellulose constructs in the gas-phase. *Green Chem.* **2022**, *24* (14), 5604-5613. DOI: 10.1039/D2GC01141G.

(12) Saito, T.; Nishiyama, Y.; Putaux, J.-L.; Vignon, M.; Isogai, A. Homogeneous Suspensions of Individualized Microfibrils from TEMPO-Catalyzed Oxidation of Native Cellulose. *Biomacromolecules* **2006**, *7* (6), 1687-1691. DOI: 10.1021/bm060154s.

(13) Winter, H. T.; Cerclier, C.; Delorme, N.; Bizot, H.; Quemener, B.; Cathala, B. Improved Colloidal Stability of Bacterial Cellulose Nanocrystal Suspensions for the Elaboration of Spin-Coated Cellulose-Based Model Surfaces. *Biomacromolecules* **2010**, *11* (11), 3144-3151. DOI: 10.1021/bm100953f.

(14) Fink, H.-P.; Purz, H. J.; Bohn, A.; Kunze, J. Investigation of the supramolecular structure of never dried bacterial cellulose. *Macromol. Symp.* **1997**, *120* (1), 207-217. DOI: 10.1002/masy.19971200121.

(15) Sheldon, R. A. The E factor 25 years on: the rise of green chemistry and sustainability. *Green Chem.* **2017**, *19* (1), 18-43. DOI: 10.1039/C6GC02157C.
